# Investigating Changes in Liver Enzyme Levels in Patients With Acute Methadone Poisoning

**DOI:** 10.1155/jt/6159057

**Published:** 2026-02-27

**Authors:** Seyed Reza Mousavi, Mahdi Arefi Ivari, Azadeh Rahmatali Khazaei, Somayeh Gharibi, Vida Vakili, Sara Rozmina, Seyed Amirhossein Mousavi, Sadaf Sadat Rafati

**Affiliations:** ^1^ Medical Toxicology Research Center, Mashhad University of Medical Sciences, Mashhad, Iran, mums.ac.ir; ^2^ Department of Community Medicine, Mashhad University of Medical Sciences, Mashhad, Iran, mums.ac.ir

**Keywords:** acute liver injury, alanine aminotransferase, aspartate aminotransferase, methadone

## Abstract

**Background:**

Today, methadone is widely used to control drug abuse. This has made this synthetic opioid widely available to the public, and its overdose has become one of the most common causes of poisoning. This study aimed to investigate changes in liver enzyme levels in patients who were referred and hospitalized due to acute methadone poisoning.

**Methods:**

This cross‐sectional study was conducted from July 2023 to June 2024 on patients hospitalized with acute methadone poisoning in the poisoning department of Imam Reza Hospital in Mashhad. Demographic and clinical information of the patients was recorded at the time of admission. Liver enzyme levels, including alanine aminotransferase (ALT), aspartate aminotransferase (AST), and alkaline phosphatase (ALP), were measured at the time of admission and before discharge. Finally, the data were analyzed using appropriate statistical tests using SPSS software.

**Results:**

A total of 49 patients were studied in this study. The mean age of the patients was 24.65 years, and 34 (69.4%) of them were female. 26% of the patients had increased AST, and 22% had increased ALT. In 80% of cases, the increase in these two enzymes was less than three times the normal limit, and no significant increase was observed. ALP was also increased in 12.2% of the patients. In total, at least one liver enzyme was increased in 38.8% of the patients. No significant correlation was observed between the dose of methadone consumed and the levels of liver enzymes (AST, ALT, and ALP) (*p* > 0.05). In addition, the increased levels of enzymes returned to normal levels during hospitalization.

**Conclusion:**

Elevated liver enzymes are a common finding in patients with methadone intoxication; however, we were unable to demonstrate a correlation between the dose of methadone consumed and serum levels of liver enzymes.

## 1. Introduction

Methadone, as a synthetic opioid of the diphenylheptylamine class, is physiologically and analgesically similar to opium, but does not have a euphoric effect and plays a key role in controlling drug addiction. It is widely used in many countries [1].

Methadone is taken orally and is rapidly absorbed from the gastrointestinal tract and metabolized in the liver and small intestine by CYP450 isoforms [2]. Methadone metabolism can be affected by several factors. Some of these factors include genetics, age, sex, liver disease, and drug interactions. Changes in methadone metabolism can affect the drug’s effectiveness and side effects. For example, individuals with faster metabolism may require higher doses of methadone to achieve the desired effect. In contrast, people with slower metabolisms are at higher risk of side effects and toxicity [3].

Although methadone treatment is generally safe, overdose can cause toxicity and impair memory and cognitive functions [4]. Methadone poisoning may happen intentionally for suicidal reasons, after an arbitrary dose increase in patients on maintenance therapy, or accidentally by children [5, 6]. In the United States, deaths from methadone overdose increased 5‐fold between 1999 and 2010 [7]. In the United Kingdom, methadone is also one of the most common causes of overdose deaths [8]. In Iran, while there are no precise statistics on the prevalence of methadone poisoning, studies indicate that methadone overdose is a significant problem that demands careful attention and planning [9].

Methadone toxicity can cause severe damage to various organs, including the heart, kidneys, liver, and central nervous system. The classic triad of toxicity includes central nervous system depression, respiratory depression, and miosis. The impact of methadone on liver function remains debated; some studies report side effects, while others find no evidence of liver toxicity [10]. One animal study showed that methadone significantly increased liver enzyme levels and inflammatory markers [11]. Another study found that methadone use can cause changes in liver tissue and elevate liver enzyme levels [12]. Some researchers believe this drug may lead to liver injury with a cholestatic pattern [13].

Research on methadone’s hepatotoxic effects is still limited. In response to the increasing number of methadone poisoning cases in Iran, this cross‐sectional study sought to investigate the acute effect of methadone on liver enzyme levels, specifically aspartate aminotransferase (AST), alanine aminotransferase (ALT), and alkaline phosphatase (ALP). The present study aimed to elucidate the possible hepatotoxic effects associated with acute methadone poisoning.

## 2. Materials and Methods

This cross‐sectional study was conducted on patients hospitalized in the Department of Poisoning at Imam Reza Hospital in Mashhad, from August 1402 to July 1403, and all stages of the study were supervised and approved by the Research Ethics Committee of Mashhad University of Medical Sciences (IR.MUMS.MEDICAL.REC.1401.349). The inclusion criteria for the study included all patients who were referred to Imam Reza Hospital and hospitalized due to acute methadone poisoning. The exclusion criteria included patients with a history of chronic methadone use and concurrent overdose of other drugs. During the study, patients with positive viral markers or those with structural diseases were excluded. Demographic information, including age, sex, history of addiction, underlying disease, and history of drug use, was recorded, along with information such as the date, amount, and dose of methadone used. To assess liver enzyme status, blood samples were collected upon admission. Before discharge of the patients, and to rule out structural diseases, all patients underwent an ultrasound of the liver and bile ducts. In addition, to investigate viral diseases, HBs Ag, HBs Ab, HBc Ab, and HCV Ab tests were requested on the second day of hospitalization. SPSS Version 16 statistical software was used for data analysis. Qualitative characteristics were compared between patients using the chi‐square test, quantitative findings between the two groups were compared using the independent *t*‐test, and correlation between quantitative variables was examined using the Pearson correlation test. In all tests, a significance level of 0.05 was considered.

## 3. Results

From July 2023 to June 2024, 1211 patients were admitted to the Poisoning Department of Imam Reza Hospital, of which 64 were admitted due to acute methadone poisoning. A total of 15 patients were excluded from the study due to meeting the exclusion criteria; the reasons for excluding these patients from the study included concomitant use of hepatotoxic drugs and substances, nonalcoholic fatty liver disease, daily alcohol consumption, and patients with CPK above 1000 U/L (suspicion of rhabdomyolysis and increased liver enzymes).

Of the remaining 49 patients, 15 were men, and 34 were women. The average age was 24.65 years (standard deviation: 10.39 years), with an age range of 16–64 years. Regarding employment status, 21 patients (42.9%) were students, 14 (28.6%) were self‐employed, 10 (20.4%) were housewives, and 4 (8.1%) were employees.

Most patients had no underlying medical conditions. However, 21 had a history of previous hospitalization for suicide or poisoning. In the assessment of the level of consciousness, most patients were drowsy or confused, and the most common clinical symptoms included miotic pupils, shortness of breath, weakness and lethargy, and nausea and vomiting. In addition, 9 patients (18.4%) presented with an oxygen saturation level below 90% on admission, and 5 patients (10.9%) experienced respiratory arrest. During hospitalization, the vital signs of all patients were stable, and no deaths occurred. Table 1 shows the characteristics of the patients at the time of admission to the hospital.

**TABLE 1:** Characteristics of the patients with acute methadone poisoning at the time of admission.

**Characteristic**		**Percentage (%)**

Past medical history	Psychiatry (depression, bipolar)	3.4
	Heart disease (ischemic heart disease with high blood pressure)	1.7
	Hypothyroidism	5.2
	Asthma	3.4

Level of consciousness	Awake	18.4
	Drowsy	38.8
	Confused	34.7
	Coma	8.1

Clinical symptoms	Miotic pupils	77.6
	Dyspnea	67.3
	Weakness	49
	Nausea and vomiting	26.5
	Blurred vision	10.2
	Itching	8.2
	Seizures	4.1
	Headache	4.1
	Restlessness	2

In the initial tests, the mean AST was 59.2 U/L with a range of 10–426. Fourteen patients (26.5%) had high AST (≥ 40 U/L), among which 4 patients had an enzyme increase in the range of 40–80 U/L, 3 patients had an enzyme increase in the range of 80–120 U/L, and 2 patients had an enzyme increase in the range of 120–160 U/L. In addition, a more than 5‐fold increase in this enzyme was observed in four patients, and a 10‐fold increase was observed in one patient. The mean ALT was 58.2 U/L with a range of 7–702. Eleven patients (22.4%) had high ALT (≥ 40 U/L), among which 4 patients had an enzyme increase in the range of 40–80 U/L, and 2 patients had an enzyme increase in the range of 80–120 U/L. A more than 5‐fold increase in this enzyme was observed in four patients, and a 10‐fold increase was observed in one patient. In addition, the mean ALP level was 171.7 U/L, with a range of 85–338 U/L; 6 patients (12.2%) had high ALP levels (≥ 258 U/L). Finally, 19 patients (38.8%) had elevated levels of at least one liver enzyme.

In the statistical analysis, no significant relationship was found between the patient’s age and the increase in liver enzymes (*p* > 0.05). Also, no relationship was found between the gender of the patients and the levels of liver enzymes. The average levels of AST, ALT, and ALP in men were 78.3, 60.7, and 176.2 U/L, respectively. These values were reported in women as 50.7, 57.2, and 169.7 U/L, respectively (in all three cases, the difference between the two sexes was insignificant [*p* > 0.05]). There was also no significant relationship found between the patient’s clinical manifestations, such as decreased level of consciousness, miotic pupils, shortness of breath, nausea and vomiting, weakness and seizures, and the levels of liver enzymes at the time of admission (*p* > 0.05). The average amount of methadone consumed by the patients was 142.86 mg with a range of 20–400 mg. Table 2 examines the correlation between the methadone dose consumed by patients and the levels of liver enzymes and other laboratory findings. As can be seen, there was no significant relationship between the methadone dose consumed and the increase in liver enzymes. However, there was a significant and direct correlation between the methadone dose consumed and the levels of total (*p* = 0.029, *r* = +0.34) and direct (*p* = 0.013, *r* = +0.38) bilirubin. This shows that the higher the methadone dose consumed by patients, the higher the total and direct bilirubin levels. Table 2 examines the correlation between the levels of laboratory parameters and the methadone dose consumed by patients at the beginning of hospitalization.

**TABLE 2:** Correlation of methadone level with laboratory data in patients with acute methadone poisoning.

Laboratory parameter	Range	Mean ± standard deviation	Correlation with the dose of methadone consumed (taking into account the Pearson correlation coefficient)
AST (U/L)	10–426	59.2 ± 86.99	*p* = 0.645, *r* = −0.06
ALT (U/L)	7–702	58.22 ± 117.89	*p* = 0.554, *r* = +0.07
ALP (U/L)	85–338	171.78 ± 62.74	*p* = 0.983, *r* = −0.03
PCO2 (VBG)	21.7–74.1	50.35 ± 11.58	*p* = 0.811, *r* = −0.03
PH (VBG)	7.11–7.52	7.29 ± 0.07	*p* = 0.762, *r* = +0.04
HCO3 (VBG)	17.7–38.1	23.99 ± 3.63	*p* = 0.822, *r* = +0.03
RBC count	3.7–5.9	4.84 ± 0.47	*p* = 0.435, *r* = +0.11
Hb (g/dL)	9.1–17.2	13.72 ± 1.76	*p* = 0.671, *r* = +0.06
WBC count	3.6–38.7	14.82 ± 6.76	*p* = 0.771, *r* = +0.04
Neut (%)	38%–92%	75.77 ± 13.56	*p* = 0.929, *r* = +0.01
Lymph (%)	4%–52%	17.6 ± 11.94	*p* = 0.837, *r* = −0.03
Plt	133–442	259.29 ± 61.7	*p* = 0.536, *r* = +0.09
Na (mEq/L)	131–144	138.38 ± 2.69	*p* = 0.106, *r* = +0.23
K (mEq/L)	3–4.9	3.89 ± 0.4	*p* = 0.121, *r* = −0.22
Urea (mg/dL)	8–54	25.24 ± 10.24	*p* = 0.787, *r* = −0.04
Cr (mg/dL)	0.7–2	1.08 ± 0.26	*p* = 0.405, *r* = −0.12
CPK (U/L)	26–753	205.53 ± 171.512	*p* = 0.525, *r* = −0.09
BS (U/L)	48–380	136.93 ± 69.99	*p* = 0.661, *r* = +0.07
LDH (U/L)	180–946	435.07 ± 199.64	*p* = 0.458, *r* = −0.14
Albumin (g/dL)	3–5	3.94 ± 0.42	*p* = 0.057, *r* = +0.35
PT	12–19	14.12 ± 1.41	*p* = 0.666, *r* = −0.07
PTT	23–100	31.13 ± 5.01	*p* = 0.243, *r* = −0.19
INR	1–2	1 ± 0.11	*p* = 0.690, *r* = −0.06
Bil T (mg/dL)	0.3–2.7	0.74 ± 0.41	*p* = 0.029, *r* = +0.34
Bil D (mg/dL)	0.1–0.6	0.21 ± 0.1	*p* = 0.013, *r* = +0.38

Also, in the linear regression analysis assessing predictors of serum methadone level on admission, several clinical and laboratory variables were evaluated (Table 3). The model showed that AST and dyspnea were significant predictors of serum methadone concentration.

**TABLE 3:** Predictors of serum methadone level in linear regression.

On admission		Unstandardized coefficients		Standardized coefficients	*t*	Sig.
		B	Std. error	Beta		
3	Constant	244.498	90.678		2.696	0.011
	AST	−0.590	0.282	−0.584	−2.097	0.044
	ALT	0.265	0.207	0.373	1.282	0.209
	Direct bilirubin	232.912	134.314	0.269	1.734	0.093
	Sex	−22.103	30.220	−0.108	−0.731	0.470
	Dyspnea	−87.063	29.258	−0.445	−2.976	0.006
	Level of consciousness	10.498	16.036	0.101	0.655	0.518

Specifically, AST was inversely associated with serum methadone level (*B* = −0.590, *β* = −0.584, *p* = 0.044), indicating that higher AST levels were associated with lower serum methadone concentrations. In contrast, ALT and direct bilirubin did not show a significant association with serum methadone level.

Among clinical variables, the presence of dyspnea was significantly associated with lower serum methadone levels (*B* = −87.063, *β* = −0.445, *p* = 0.006). Level of consciousness did not show a significant relationship with serum methadone concentration (*p* = 0.518).

**FIGURE 1:**
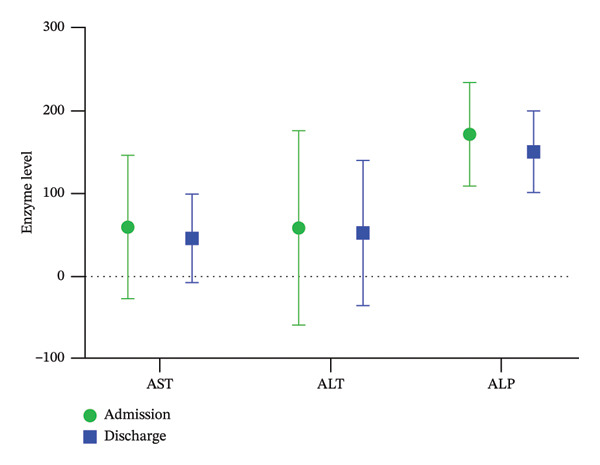
Comparison of the level of liver enzymes (mean ± standard deviation) at the time of admission with the time of discharge.

Finally, during hospitalization, most of the patients (about 90%) experienced a significant decrease in liver enzymes, and only 5 patients experienced an increase in liver enzymes. Of these, 4 patients only had an increase in ALT, which could be due to a later increase in this enzyme than in AST and a later decrease. However, eventually, these people also had enzyme reduction. Table 4 demonstrates the levels of liver enzymes at three time points after methadone administration (on admission to hospital, Day 2 after admission, and discharge) for males, females, and the total population. In addition, Figure 1 shows a comparison of the difference in liver enzyme levels at admission and at discharge.

**TABLE 4:** Liver enzyme concentrations by time.

		AST (mean ± SD)	ALT (mean ± SD)	ALP (mean ± SD)
Admission	Male	78.33 ± 114.02	60.53 ± 86.83	176.27 ± 54.46
	Female	50.76 ± 72.47	57.21 ± 130.44	169.79 ± 66.74
	Total	59.2 ± 86.99	58.22 ± 117.89	171.78 ± 62.74

Day 2	Male	60.00 ± 61.31	67.60 ± 84.10	—
	Female	31.00 ± 12.68	49.00 ± 44.23	—
	Total	39.87 ± 27.56	54.69 ± 56.43	—

Discharge	Male	52.07 ± 62.10	44.87 ± 50.53	150.80 ± 41.21
	Female	42.65 ± 50.29	55.29 ± 101.04	150.50 ± 53.40
	Total	45.53 ± 53.69	52.1 ± 88.24	150.6 ± 49.38

## 4. Discussion

The present study aimed to demonstrate the relationship between serum levels of liver enzymes (as a marker of liver damage) during acute methadone intoxication. More than one‐third of the patients included in our study had at least one liver enzyme elevated. It should be noted that we excluded patients with other known causes of elevated liver enzymes (such as fatty liver or viral hepatitis). Of the patients with elevated serum enzyme levels, the pattern of elevation was hepatocellular in most cases (meaning that the elevation in AST and ALT levels was more pronounced than the elevation in ALP levels). In most of these patients, liver enzyme levels decreased during hospitalization and approached the normal range.

Overall, these results underscore the complexity of factors influencing serum methadone levels in poisoned patients and suggest that biochemical markers of liver function and respiratory symptoms may provide complementary information beyond serum drug concentrations alone. Further studies with larger sample sizes and longitudinal measurements are needed to clarify the underlying mechanisms and clinical implications of these associations.

Elevated liver enzymes have been reported in previous studies with methadone. In an animal study by Amraei et al. in 2018, serum liver enzyme levels were significantly increased in male rats that received methadone for 8 weeks compared to a group that did not receive methadone [10]. Another animal study by Zaligder et al. in 2023 showed that methadone treatment in rats can increase liver enzyme levels compared to rats that did not receive methadone [14]. Human studies are very limited. In one such study, conducted by Eslami‐Shahrbabaki et al. in 2012, liver enzyme levels were increased in a small number of patients who received a maintenance dose of methadone for 2 years, and the observed increase had a cholestatic pattern [13]. The important difference between our study and the aforementioned studies is that our study focused on patients who had acute methadone intoxication and excluded patients who were chronic methadone users. However, considering that the mentioned studies have also reported an increase in the level of enzymes, it can be concluded that the use of methadone, either with a low dose and for a long time or with a high dose acutely, can lead to changes in the levels of liver enzymes. However, the study by Eslami‐Shahrbabaki et al. indicated the presence of a cholestatic pattern in patients who had used methadone for 2 years, while in our study, acute high‐dose methadone use was associated with a hepatocellular pattern in most patients [13].

Several hypotheses have been proposed regarding the cause of liver injury following methadone use. Excessive methadone use may increase free radical production and decrease antioxidant defense systems in the liver, which may result in liver cell damage and elevated liver enzyme levels [15]. In addition, methadone may affect cellular processes such as mitochondrial respiration and energy production in liver cells, impairing cell function [16] or leading to programmed cell death (apoptosis) of liver cells [17, 18]. Finally, methadone may increase inflammatory cytokines and stimulate an inflammatory response in the liver [19].

Another important finding of our study was that no significant correlation was found between the dose of methadone used by the patients and the severity of the increase in liver enzymes; in other words, some patients with low doses had a significant increase in enzyme levels, while in those who took high doses, there was no significant increase in enzyme levels. Of course, there was a significant and direct correlation between serum bilirubin levels in patients with increased liver enzymes, although in this case, too, the observed correlation was weak (correlation coefficient less than 0.4). In interpreting this finding, it can be stated that liver injury in patients with methadone poisoning may be idiosyncratic, meaning that there is no linear relationship between the severity of liver injury and the dose of methadone used. However, unlike our study, in the animal study of Amraei et al. [10], which was mentioned earlier, there was a significant relationship between the increase in the serum level of liver enzymes and the dose of methadone. However, it should be noted that various factors may have confounded the results and led to such a result, including the fact that patients may not have accurately reported their methadone dosage.

Our study, like other studies, had strengths and weaknesses that need to be considered. One of the weaknesses of the present study was its cross‐sectional nature, which makes it difficult to establish a cause‐and‐effect relationship between methadone use and increased enzyme levels, and therefore, we can only conclude that there is a relationship between them. Also, since we asked the patient about the dose of methadone used, it is possible that the methadone dose that the patient reported was not true. On the other hand, the strengths of our study include the exclusion of patients with liver disease and patients with a history of taking medications that affect liver enzymes.

## 5. Conclusions

Elevated liver enzymes are a common finding in patients with acute methadone intoxication; however, these elevations return to normal within 3 days. On the other hand, there is no correlation between the dose of methadone consumed and the serum level of liver enzymes. These findings can help improve physicians’ understanding of the effects of methadone on liver function and related treatment approaches.

## Author Contributions

Seyed Reza Mousavi and Somayeh Gharibi: conducted the main idea of the study and supervised the study.

Vida Vakili: data analysis.

Mahdi Arefi Ivari: drafting of the manuscript.

Azadeh Rahmatali Khazaei and Sara Rozmina: data gathering and recording.

Seyed Amirhossein Mousavi: revised the manuscript.

Sadaf Sadat Rafati: revised the manuscript (corresponding author).

## Funding

The present study was funded by the Mashhad University of Medical Sciences, Mashhad, Iran.

## Ethics Statement

The method has been approved in terms of compliance with scientific and ethical standards of evaluation. All methods were performed in accordance with the relevant guidelines and regulations. The Organizational Ethics Committee of Mashhad University of Medical Sciences, Mashhad, Iran, has also approved it (IR.MUMS.MEDICAL.REC.1401.349). Patients filled out a written informed consent before participating in the study. In addition, no extra costs have been incurred by patients for this study, and patients have been studied during their treatment and diagnostic process.

## Consent

Please see the Ethics Statement. No personal identifying details will be published.

## Conflicts of Interest

The authors declare no conflicts of interest.

## Data Availability

The data that support the findings of this study are available on request from the corresponding author. The data are not publicly available due to privacy or ethical restrictions.
